# Smart Fluid Systems: The Advent of Autonomous Liquid Robotics

**DOI:** 10.1002/advs.201700036

**Published:** 2017-06-22

**Authors:** A. Chiolerio, Marco B. Quadrelli

**Affiliations:** ^1^ Center for Sustainable Future Technologies Istituto Italiano di Tecnologia Corso Trento 21 10129 Torino Italy; ^2^ Jet Propulsion Laboratory California Institute of Technology Pasadena CA 91109‐8099 U.S.A.

**Keywords:** colloidal robotics, colloids, liquid devices, liquid engineered systems, smart fluid systems

## Abstract

Organic, inorganic or hybrid devices in the liquid state, kept in a fixed volume by surface tension or by a confining membrane that protects them from a harsh environment, could be used as biologically inspired autonomous robotic systems with unique capabilities. They could change shape according to a specific exogenous command or by means of a fully integrated adaptive system, and provide an innovative solution for many future applications, such as space exploration in extreme or otherwise challenging environments, post‐disaster search and rescue in ground applications, compliant wearable devices, and even in the medical field for in vivo applications. This perspective provides an initial assessment of existing capabilities that could be leveraged to pursue the topic of “Smart Fluid Systems” or “Liquid Engineered Systems”.

## 1 Introduction

Extreme planetary environments represent the next frontier for in‐situ robotic space exploration, where reconnaissance missions will be followed by robotic in‐situ missions, and perhaps later by human exploration. All these missions have one common problem: harsh, extreme environments, where temperature, radiation, and other factors making many of these missions inconceivable at present. Extreme environments, on Earth and other locations in the Solar System, are characterized by low or high temperatures, high‐radiation, high pressure, corrosive and toxic chemicals, etc. As scientific knowledge advances, autonomous missions of increasing complexity are needed to negotiate extreme environments, and these place increasing demands on the corresponding autonomous robotic platform technologies.^1^

Compared to conventional robotic systems (i.e., anything not liquid), colloid‐based robotic systems offer enormous promises, in terms of versatility, adaptability, resiliency, distributed architecture, and autonomy, but have not yet been explored at length.^2^ A soft and deformable robot would be a desirable platform for traversing unpredictable terrain, navigating through small holes, or even for interacting with humans where unintentional infliction of harm is of great concern. This brings along future applications in the exploration of harsh environments, for example gas giant planets or small bodies like comets and asteroids, oceans and lakes depths, rough terrains, post‐earthquake areas, surgery, and so on. Scope of this perspective review is to report on the advancement and state‐of‐the‐art of materials science and robotic systems technology in the field of Smart Fluid Systems (SFS)/Liquid Engineered Systems (LES) that could lead to further research and technology development of liquid robotic systems in the near future.

### 2 Soft Robotics versus Colloidal Robotics

Soft Robotics represents a well established and fastly growing field of current research, where the robots are conceived as easily deformable objects, based on fluids, gels, and polymers that mimick the mechanical and rheological properties of their biologic counterparts achieving the required compliance matching.^3^ This point of view represents a strikingly different vision from the traditional hard, solid, articulated and heavy robots operating in industrial environments with which everyone is familiar, and promises to be even more sustainable, from the energetic point of view.^4^, ^5^ SFS based on colloids woulc offer a change in paradigm, since the physics behind nanostructured fluids, the interactions among their constituents such as molecules and particles on one side, and their collective properties on the other side, would make them a completely different system, and justify the need to separate Colloidal Robotics from Soft Robotics.^6^ Furthermore, colloid properties under equilibrium differ completely from the same properties of the so called “active colloids”, kept out of thermodynamic equilibrium by external forces such as electric, magnetic, gravitational, thermal fields. Self‐assembly and other specific phenomenologies would emerge in such conditions, making it possible to control and operate SFS from a distance.^7^

## 3 Definitions

A list of selected keywords is defined here for the sake of the reader:

- **Liquid** is a condensed matter state in the solid state, featuring no shape retention but almost perfect volume retention, if submitted to pressure variation.
- **Fluid** is a condensed matter state in which shear forces result in a flow under stress without compromising the continuum of the object; both liquids and gases are fluids.
- **Colloid** is a complex condensed matter system lying at the boundary between completely homogeneous systems such as solutions and completely heterogeneous systems such as suspensions. Generally, they are composed of a fine phase dispersed in a solvent in liquid or gas form. They are classified according to the aggregation state of their components (S = solid, L = liquid, G = gaseous): S‐S (solid sol), S‐L (solid emulsion), S‐G (solid foam/aerogel), L‐S (sol), L‐L (emulsion), L‐G (liquid foam), G‐S (solid aerosol), G‐L (liquid aerosol), G‐G, where the first letter refers to the dispersant (carrier phase) and the second to the dispersoid (suspended phase). Colloids feature non‐trivial collective properties and can present two phase transitions: *flocculation*, occurring when the dispersoids coalesce, and *gelation*, occurring when the dispersant changes viscosity. Those phase transformation can be reversible, and can also depend on an exogenous input for that reversibility to occur.
- **Filler** or **dispersoid** is a solid (nano‐ or micro‐) particle with a different chemophysical nature with respect to the carrier (liquid solvent or gaseous phase).
- **Smart fluid** (SF) is a liquid‐based device with collective properties endowed by the cooperation of the dispersoid and dispersant phases, whose single agents or constituents enable the emergence of smart distributed functionalities such as information processing capabilities, self‐powering, sensing capabilities, and mobility.
- **Ferrofluid** (FF) is a colloidal suspension of NPs (nanoparticles) in the superparamagnetic (SPM)/ferromagnetic (FM) state, capable of developing long‐range attractive interactions that are compensated by short range repulsive interactions due to steric hindrance, given by particle functional groups.
- **Magneto(electro)rheological fluid** (M(E)RF) is an unstable liquid phase, a ferromagnetic(dielectric) suspension that could be stabilized by the external application of a magnetic(electric) field and formed as a result of magnetization(polarization) forces.
- **Superfluid** refers to a quantum mechanical condensed matter state where its atomic constituents behave as bosons in the condensed state, and could be conditioned to have collective properties by an external action.
- **Supercritical fluid (SCF) refers to any liquid above its triple point**.

## 4 Assumptions for a Scale‐Dependent Approach

**Figure 1:**
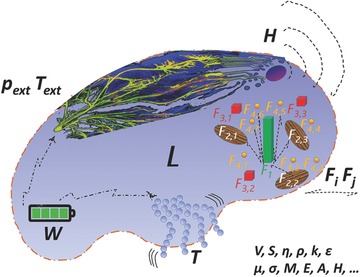
System Concept. Artistic view of the system concept typical for a SFS of macroscopic volume *L*, including different fillers whose interaction matrix is *F*_i_ *F*_j_, enabling a number of basic functionalities such as energy (*W*) storage and distribution, mobility (*T*) and information storage, processing and remote transmission (*H*). Physical transduction is achieved through a variation of one or more properties: volume *V*, shape *S*, viscosity η, density ρ, thermal conductivity *k*, dielectric constant ε, magnetic permeability *µ*, electrical conductivity σ, magnetization *M*, emissivity *E*, absorbance *A*, information entropy *S*.

In an attempt to develop an approach to address the SFS feasibility, we make the following scale‐dependent assumptions (**Figure** **1**).

### 4.1 Macroscopic Scale

We assume that a certain volume of the SFS, function of the characteristic length *L*, is positioned in a desired location on a desired environment, having pressure (*p*_ext_) and temperature (*T*_ext_), possibly protected by a flexible skin, such that evaporation/freezing of the same fluid are controlled, as well as collective phase transformations, or decoherence of either single agents or eventual dispersed phases/fillers. *L* is a characteristic size in the macroscopic domain, anywhere in the range 1 mm < *L*_M_ < 1 m. It would be possible to actively interact with *L* and induce a modification of its viscosity, aggregation state, internal state, volume and shape. Furthermore the external environment would induce modifications in the same way. After changing the state at the macroscopic/collective level *L*, the SFS would be able to transfer information back to the external environment according to a pre‐planned program.

### 4.2 Mesoscopic Scale

Within the scale *L*, the volume would feature an internal structure such that smart functionalities could be enabled by triggering its state externally (either from a deliberate actuation mechanism, or from the environment). Smart functionalities would depend on the degree of interaction at the mesoscale of smaller elements *F* (single agents or more specifically either micro or nanostructured fillers, or a combination of the two) between themselves (*F*_i_ *F*_j_) and with the liquid matrix (*F*_i_ *L*). The mesoscale domain takes place at the characteristic size, anywhere in the range between 10 nm < *L*_m_ < 1 mm. Any physical transduction at this level would be considered a smart functionality, in particular: volume *V*, shape *S*, viscosity η, density ρ, thermal conductivity *k*, dielectric constant ε, magnetic permeability *µ*, electrical conductivity σ, magnetization *M*, emissivity *E*, absorbance *A*, information entropy *H* variations are all envisaged to lead to a transduction of an environmental modification.

### 4.3 Nano/Microscale

At this scale, *L* and its internal structure would be such that a subsystem of basic functionalities could be provided, in particular: energy (*W*) storage/generation/distribution; information (*H*) collection/storage/distribution/elaboration/transmission; mobility (*T*). This microscopic domain has a characteristic size anywhere between 100 pm < *L*_µ_ < 100 µm. The distribution of energy and information could involve diffusion/drift/ballistic transport/hopping of electrical charges (electrons, holes, ions, charged clusters of atoms), of magnetic moments (electrons, holes, ions, magnetic clusters of atoms, nanoparticles (NPs), microparticles (µPs)), of vibrations (phonons, acoustic waves), of electromagnetic waves (photons) either through the liquid or through physical connections floating in the liquid. The sensing capabilities could involve fillers in the form of autonomous nano‐ or micro‐particles (such as smart dust, or similar), in other words complex MEMS devices of small enough size not to collide with the mesoscopic and macroscopic assumptions, possibly wired in. Mobility could also involve diffusion/drift of chemical species in any form, but also could be due to mesoscopic or macroscopic level actions taking place in the smart fluid.

## 5 Materials

To realize SFS, a variety of liquids enabling the function of synthetic cytoplasm could be used: ionic liquids, low melting point metals, polar/nonpolar solvents, inorganic liquids, acting all as solvents to disperse functional fillers. Colloids, in particular FFs and M(E)RFs, would also be important, as well as metal liquid‐like films (MeLLFs).

### 5.1 Ionic Liquids

**Figure 2:**
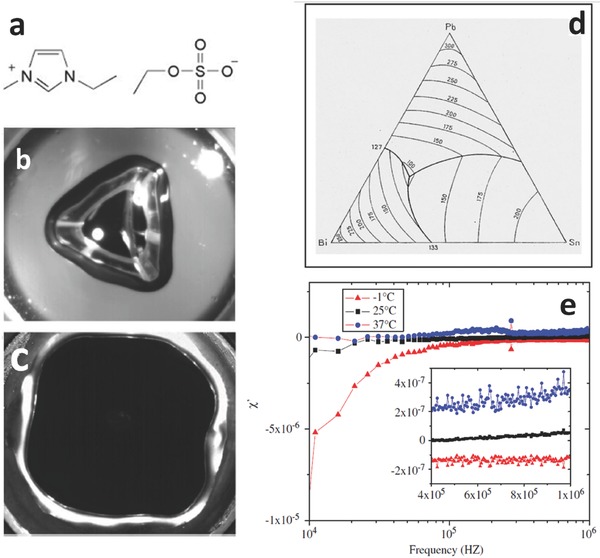
Liquid Matrices. a) formula of the [emim][EtOSO_3_] IL. b) observation of drop oscillations through high‐speed camera in liquid Galinstan under cyclic magnetic field, generated forcing a current into an inductor at 20 kHz. The system is characterized by the Bond number (dimensionless parameter being the ratio between gravity force and surface tension force) *Bo = 22*, and the mode number *m = 3* featuring triangular symmetry. c) same as before with *Bo = 39* and *m = 4* featuring square symmetry. b,c) Reproduced with permission.^13^ Copyright 2006, APS. d) phase diagram of the Bi/Pb/Sn system showing the position of the ternary eutectic melting at 96 °C, compositions are in weight percentages. Reproduced with permission.^14^ Copyright 2010, ACS. e) susceptibility of water at −1, +25 and +37 °C, showing a transition of the polar solvent between paramagnetism to diamagnetism across 25 °C. Reproduced with permission.^15^ Copyright 2012, Elsevier.

Ionic Liquids (ILs) are salts which are liquid below the boiling temperature of water, are based on ionic structures, very frequently having low vapor pressures at room temperature or below, normally featuring a high viscosity and potentially could be either hydrophobic or hydrophilic.^8^ As an example, 1‐ethyl‐3‐methylimidazolium ethylsulphate (EtOSO_3_) has a melting point of 175 K. Furthermore they are non‐flammable, and commonly thermally stable over a wide range of temperatures.^9^ This would make them a suitable candidate for future space grade lubricants that seldom operate at temperatures lower than 230 K.^10^ More recently energetic ionic liquids have been synthesized and proposed as propellant.^11^ These liquids could be used pure or in binary/ternary compounds, associated to other solvents.^12^ Over a vast list of more than 500 ILs, making a selection in terms of non‐hazardousness, hydrophobicity and melting temperature below 250 K, results in approximately 30 different products. Considering then the ASTM E595 outgassing test, typically performed to determine evaporation characteristics of the materials on the basis of Total Mass Loss (TML, loss of a material subjected to 398 K at p < 7 × 10^−3^ Pa for 24 h) and Collected Volatile Condensable Materials (CVCM, capability to condense on a collector at 298 K), only seven ionic liquids were found useful, based on TFSI (triFluoroMethyl)Sulfonylamide anions. In particular, 1‐ethyl‐3‐methyl imidazolium ethylsulfate known as [emim][EtOSO_3_] (**Figure** **2**a) has no toxicity issues, molar mass 236.29, melting point below 250 K and a density of 1.24 g cm^−3^ at 350 K.

### 5.2 Low Melting Metal Alloys

Low melting metal alloys normally have a melting temperature below 500 K, and generally contain Ga, or Sn, or Pb, or Bi. For instance, Galinstan (Ga 68% In 21% Sn 10% Sb 0.5% Bi 0.5%, though there are controversies regarding their effective composition and melting point), a trademark produced by Geratherm Medical AG, is said to have a melting point of 254 K.^16^ Molten metals are attractive since an electromagnetic field could produce hydrodynamic instabilities and shape them (Figure 2b,c).^13^ Electromagnetic shaping of free surfaces of metal liquids is a well‐known technology currently applied in metallurgy.^17^ Eutectic alloys undergo a reversible non‐hysteretic melting, while non‐eutectic alloys have a melting range instead.^14^ As an example alloy Cerrolow 117 (Bi 44.7% Pb 22.6% Sn 8.3% Cd 5.3% In 19.1%) has a melting point of 320 K, making it suitable for liquid applications in the Venus environment (Figure 2d). Liquid metals in the form of low melting alloys have started to be applied in the field of autonomous systems.^18^

### 5.3 Polar Solvents

Polar solvents represent the most diffused, less expensive, matrix available nowadays for a SFS, in particular water. They are characterized by not perfectly understood effects induced by high electric/magnetic fields, such as the reduction of the strength of hydrogen bonds, which is likely to occur in a FF.^19^, ^20^, ^21^ Regarding water, the most relevant effect we may point out is the change of magnetic susceptibility from negative (diamagnetism) to positive (paramagnetism), depending on temperature and frequency (Figure 2e).^15^ This effect could be exploited to induce attracting/repelling behavior, changes in volume and shape, in temperature‐controlled, water‐based SFS. Regarding other compounds, we can point out that certain esters and alcohols can have an extended working range, between the melting and the boiling point, up to 300 K, showing good thermal stability.

### 5.4 Nonpolar Solvents

Nonpolar solvents can be divided into aliphatic, aromatic and halogenated. Aliphatic ones have the lowest densities among all solvents (including polar), which could be a point of interest in situations requiring mass constraints. This would be counterbalanced by their higher evaporation rate.^22^

### 5.5 Inorganic Solvents

Inorganic solvents would include chemicals such as carbon disulfide CS_2_ and carbon tetrachloride CCl_4_ (toxic), phosphorus tribromide PBr_3_ (may evolve toxic HBr or explosive phosphine), sulfuryl chloride fluoride SO_2_ClF (boils at 240 K, moderately toxic), dinitrogen tetroxide N_2_O_4_ (hypergolic and toxic), antimony trichloride SbCl_3_ (melts at 346.5 K, dangerous) could be also used as matrices, even though their toxicity does not make them the optimal choice.^22^

### 5.6 Metal Liquid‐Like Films

**Figure 3:**
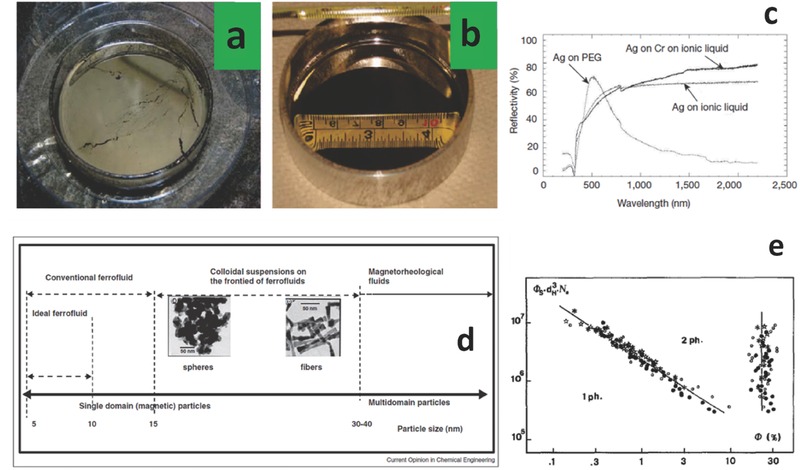
Nano colloids: MeLLFs and FFs. a) Magnetically deformable liquid mirrors MeLLFs prepared from Ag NPs spread on the surface of ethylene glycol based FFs containing Fe oxide NPs coated with citrate. b) MeLLFs‐based liquid mirror prepared with Fe oxide NPs coated with 2‐[2‐(2‐methoxyethoxy)ethoxy]acetic acid (MOEEAA). Reproduced with permission.^25^ Copyright 2008, ACS. c) Reflectivity curves obtained for silver‐coated MeLLFs. The best performance at low wavelengths was achieved coating a hydrophilic block copolymer (PEG), while the best toward infrared was obtained coating ILs. Reproduced with permission.^24^ Copyright 2007, Nature Publishing Group. d) magnetic fluid classification, highlighting the boundaries between FFs and MRFs. Reproduced with permission.^27^ Copyright 2014, Elsevier. e) bilogarithmic plot of the reduced phase diagram valid for any FF sample. The dots represent different samples, the full lines a model highlighting the boundaries between monophasic and diphasic regions. Reproduced with permission.^28^ Copyright 1989, Elsevier. Φ_S_ is the ionic strength normalized to the global volume fraction of magnetic NPs in the system, *d*_H_ is the NP diameter determined through characteristic time measurement of birefringence relaxation, *N*_a_ is Avogadro's number and ϕ is the volume fraction of NPs.

Metal Liquid‐Like Films (MeLLFs) consist in interfacial layers of typically silver NPs trapped at the interface between two immiscible liquid phases, for example a polar (water) and an apolar (dichloroethane) one, or between air and liquid interface.^23^ Due to plasmonic resonance and interaction between adjacent particles, MeLLFs show both optical reflection and macroscopic fluidity (**Figure** **3**a,b and c).^24^ In regards to the realization of an orbital/lunar adaptive telescope, it has been reported that silver based MeLLFs are not compatible with hydrocarbon based FFs, but rather require an ionic FF, stabilized by electrostatic charges.^25^ Regarding toxicity, Ag NPs are well known to have antibacterial properties, therefore their in vivo application could pose some issues.^26^ Above certain levels, exposure to Ag ions and NPs could be poisonous and induce a syndrome called argyria (vampirism), a non‐life threatening condition (above 5 µg/kg/day intake).

### 5.7 Ferrofluids

The typical constituents of FFs are: γ‐Fe_2_O_3_, Fe_3_O_4_ or other ferrites (for example Co‐based), MeFe_2_O_4_ (spinels, where Me = Mn, Co, Zn, Ni, and so on), ε‐Co, Fe_.75_Co_.25_ dispersed in water or ethanol or kerosene. The synthesis of magnetic NPs follows the same routes used for the synthesis of other types of NPs: top‐down approaches where a bulk is broken by mechanical action into smaller particles until the desired size distribution is reached (high‐energy ball milling represents the best example) and bottom‐up approaches, where chemical reactions nucleate and grow the particles up to the desired size distribution (gas phase methods, spray pyrolysis methods, inert gas condensation, liquid phase methods, hydrothermal synthesis, sol‐gel synthesis and microemulsion method).^29^ To prevent dispersoid aggregation due to London‐van der Waals and magnetic interactions, the particles are bound to surfactants, for example oleic acid or tetramethylammonium hydroxide. The size of the magnetic particles ranges between 5 and 40 nm but would strongly depend on the nature and shape of the fillers.^27^ Reported operating temperatures range between 5 and 125 °C, their typical viscosity ranges between 50 and 500 cP, and their stability is confirmed up to 1,000 g. They are currently used as moving seals, moving sensors, in high fidelity loudspeakers, as inertial dampers and in the oil industry.^30^ Others have proposed solutions at the smooth boundary between FFs and MRFs (Figure 3d).^27^

**Figure 4:**
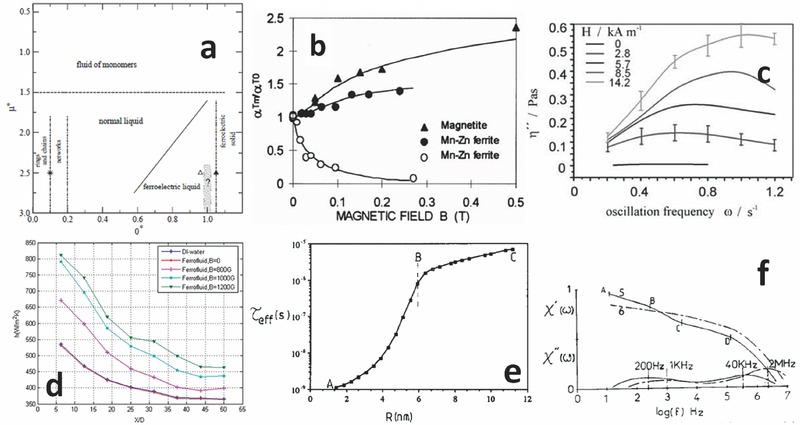
FFs physico‐chemical properties. a) Phase diagram obtained according to the Discrete Hard Sphere (DHS) 3D model in the reduced temperature – density plane. The reduced temperature is related to the reduced dipole moment T* = µ* ^−^ *^2^*. At low temperatures an association of particles in chains and rings is seen; increasing the density increases the average cluster size, and then results in a normal liquid condition. Further compression brings to a ferroelectrically ordered phase. The start indicates a critical point transition conjectured, the triangles indicate the limits of mechanical stability of body centered tetragonal (open) and face centered cubic (filled) solid phases. Reproduced with permission.^31^ Copyright 2005, Elsevier. b) magnetic Soret effect in tetradecene based FF stabilized by oleic acid, magnetic field perpendicular to temperature gradient (filled dots) or parallel to it (open dots). Reproduced with permission.^32^ Copyright 1999, Elsevier. c) Magnetoviscoelastic effect: imaginary part of complex viscosity of a commercial FF under different magnetic field strengths. Reproduced with permission.^33^ Copyright 2003, Elsevier. d) heat transfer coefficients and effects of the thermomagnetic convection. Reproduced with permission.^34^ Copyright 2010, Elsevier. e) Effective relaxation time of a magnetic FF as a function of NP radius. f) experimental normalized plots of real and imaginary components of magnetic susceptibility as a function of frequency logarithm for two FF samples. Reproduced with permission.^35^ Copyright 2005, Springer.

Depending on the nature of the interaction between filler and solvent, we could separate the FFs in different cathegories: IM (ionic FFs having unbalanced charges and electrical conductance) and M (purely magnetic fluids).^30^ Depending on the nature of the filler, we can distinguish between monodomain ferromagnetic particles (FM) and superparamagnetic particles (SPM), with a critical particle size that separates the two regimes. The phase diagram is rather complex (Figure 3e), since temperature, magnetic field and ionic strength can influence the aggregation status of the FF; in particular increasing the magnetic field is equivalent to increasing the ionic strength or to lower the temperature (**Figure** **4**a).^31^ A transition between liquid and gas phase was predicted, and associated to a reversible phase separations in the IM, between more concentrated droplets and less concentrated media.^28^ This phase transformation/separation, experimentally observed, is important: the highly concentrated fluid has a composition around 24% of particles with a low volume content of solvent and features a very low interfacial tension, allowing to support hydrostatic instabilities. However phase diagrams are particle‐size‐distribution dependent, in particular they are correlated to a high order (≈3) moment of the particle size distribution. Dimers, trimers, chains of particles start to form and stabilize when the dipolar energy is larger than the thermal one.

Under nonequilibrium conditions two mutual effects were measured in FFs: the Soret and Dufour effects, in other words a thermal induced diffusion of species that tends to separate two components of different masses in a fluid, and a heat flow that is exchanged between segregated components.^36^ The thermal mobility is relevant for such small NPs as in FFs. It is extremely difficult to separate using useful Soret effects, in an experimental setup to measure coefficients, from other spurious effects, such as viscosity changes and gravitational effects. Interestingly, the magnetic field affects the diffusion of particles depending on the orientation with respect to the temperature gradient, so that it is possible to concentrate particles both in the colder and in the hotter spots (Figure 4b).^37^ Ultimately, this involves a non‐potential force field: a non‐uniform magnetic field, in the presence of a thermo‐gravitational field, in a nonhomogeneous medium such as a FF, when the NPs feature a susceptibility function of both magnetic field and temperature (pyromagnetic coefficient), results in the so called Kelvin body force, that produces the thermomagnetic convection.^32^, ^34^ This field of research seems to have expanded exponentially in the last years, due to various practical needs of increasing the heat transfer efficiency in computing devices (Figure 4d).^38^ In regards to more conventional uses of viscosity in FFs, monitoring its imaginary component allows evaluating the relaxation time of complex structures induced by the external magnetic field (Figure 4c).^33^, ^35^ The electromagnetic properties of M FFs depend on two relaxation mechanisms: one is the Brownian motion, the other is Néel electromagnetic relaxation. A regime in which the latter prevails is found at small hydrodynamic radii (r < 6 nm), while the former prevails for bigger particles. By using a toroidal arrangement, it would be possible to measure the complex permittivity of FFs, in AC fields as well as adding a DC magnetic field. The corresponding Debye profiles of permittivity show a characteristic absorption peak in the imaginary component, that easily allows the determination of the corresponding relaxation time and characteristic radius (Figure 4e,f). By adding a DC component in the magnetic field, it would be possible to observe a resonance due to the precession of magnetic moments, described by Landau – Lifshitz equations.^39^

Cryogenic industrial application of FFs are not known yet, but have already been thought of.^40^ Normally commercial products have a pour point ranging from 180 to 250 K. Magnetic properties of the constituent NPs are kept well below the blocking temperature, generally in the range between 5 and 25 K, meaning that it could be possible to create a cryoferrofluid (CFF) using particular solvents. Such an SFS would find applications in the cryogenic environment at the surface of Titan.

Regarding toxicity, we also point out that any compound containing Co or Ni is toxic, if not cancerogenic. Magnetite is very safe in handling and also biocompatible.^41^ For simple experiments and non‐optimized magnetohydrodynamic properties, magnetite would represent the best choice.

### 5.8 MagnetoRheological Fluids

**Figure 5:**
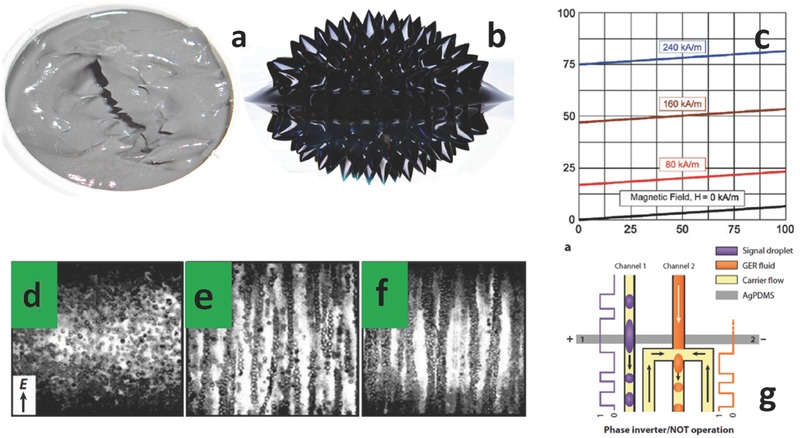
Micro colloids: MRFs and ERFs. a) MRF from Lord Corp. b) FF from Apexmagnets LLC. c) effect of magnetic field strength on the shear yield stress of a MRF. Reproduced with permission.^43^ Copyright 2013, Gruppo Italiano Frattura. d) morphological evolution of a ERF composed by glass dielectric microspheres suspended in silicone oil under no external electric field. e) same as before with external electric field of 500 Vmm^−1^. f) same as before with external electric field of 900 Vmm^−1^. Reproduced with permission.^44^ Copyright 2008, RSC. g) basic working principle of a microfluidic logic gate based on GER fluids: when a signal droplet (conductive) passes through the signal electrodes, the GER fluid is solidified and stopped, while carrier oil keeps flowing. Hence, the sequence of GER droplets has a phase which is complimentary to that of the signal droplet, having realized an inverter (NOT Boolean operation). Reproduced with permission.^45^ Copyright 2010, RSC.

MRFs show a sharp variation of their non‐Newtonian behavior, increasing their viscosity upon the application of an external magnetic field. The effect is reversible and the typical response time of commercial materials is in the 5 ms range. Application fields range from dampers in the automotive compartment, anti‐earthquake systems, body bullet‐proof armor military systems. Differently than FFs, the solid loading can be as high as 90% and the typical ferromagnetic filler size is in the range 1–10 µm (**Figure** **5**a,b and c). Under strong gravitational fields, therefore, those liquids experience sedimentation.^42^, ^43^

### 5.9 ElectroRheological Fluids

ERFs present the same properties as MRFs, but enabled by submitting them to a high electric field (above 1 kV mm^−1^). They are based on coated spherical metal oxide particles, such as BaTiO_3_, PZT, TiO_2_, typically dispersed in silicon oil (Figure 5d to f). Optimized materials led to the realization of so called Giant ER fluids (GER), currently under study for the realization of liquid logic devices, where the binary code is interpreted in terms of GER (water) droplets for 0 (1), in a sequence electrically controlled in microfluidic devices (Figure 5g).^44^, ^46^

Comparing the two fluids, the operational temperature range is slightly wider for MRFs (230–420 K) than for ERFs (245–395 K), while the power demand is the same (1–50 W), even though to generate a high strength magnetic field and operate a MRF (≈ 1 T) a heavy equipment is required (either permanent magnets or copper coils, magnetically soft cores and a high power generator), while to operate an ERF system a much lighter equipment is required (a voltage multiplier is a solid state device quite simple and small). Their application in SFS could lead to enable mobility applications through volumes of variable stiffness.

### 5.10 Diverse Fillers

Besides the basic colloids (MeLLF/FF/MRF/ERF), enabling vital functionalities such as sensing and mobility, several different fillers could be added to the SFS to achieve additional functionalities or augment functionalities already in place. In particular, the following components could be added:

- Discrete **MicroElectroMechanical Systems** (MEMS) and/or **Integrated Circuits** (ICs) allowing the distribution within the SFS liquid matrix of a number of circuits for information storage and processing, MEMS tools to induce vibrations in the SFS through piezoresonators, integrated solid state lasers/phototransistors for local measurements, integrated Hall effect magnetic sensors, thermistors as temperature sensors, piezoelectric systems as pressure sensors, and so on. ICs could also integrate their own antenna for communication in certain bands, as well as supercapacitors/batteries for energy storage, enforcing also ultra low power functionalities.^47^
- **Functional µPs** (microparticles) would enable the tuning of the SFS physical properties, such as dielectric constant, global magnetization, refraction index, and so on. This could be achieved using either full particles, or hollow ones, or even complex structures such as mycellae.
- **Functional NPs** (nanoparticles). Besides the NPs composing the FF or the MeLLF, one could add other NPs having SPM, super‐paraelectric, or other different features, such as fluorescent quantum dots.^48^
- **Diverse Aspect Ratio Fillers,** including wires, rods, tubes, platelets, ribbons, scrolls, tetrapods, cubes, each having peculiar properties that could be helpful to further tune the SFS physical properties.^49^

A fundamental aspect would be that of functionalization, in order to make the dispersoids compatible with the liquid matrix of the SFS, and provide correct stability towards gravitational precipitation, chemical reaction, and so on.

## 6 System Functionalities

To provide the required functionalities that would enable practical applications, the SFS could leverage the collective properties of its constituents, relying on the variation of the following physical properties: Shape *S* variation, Viscosity η variation, Thermal conductivity *k* variation, Permittivity ε variation, Permeability *µ* variation, Conductivity σ variation involving charge transfer processes, Magnetization *M* variation, Absorbance *A* variation, and Information entropy *H* variation, among other possible ones.

Next, we address the capabilities of SFS in the areas of energy generation, energy storage, information collection, storage, and transmission, sensing, and mobility.

### 6.1 Energy Generation

Considering the SFS as a living complex system, we could think of adding homeostatic functionalities to enable stable operation in safe conditions. This could be done either using active devices (Joule heaters for example, controlled by integrated circuits – ICs) or by using passive systems able to release phase‐transformation enthalpy and help in maintaining a constant temperature over a temperature range. Many of those devices are already commercially available, both for high‐tech outdoor applications, and nowadays for energy saving/management in residential buildings and offices. Commercial products are already able to release up to 200 kJ kg^−1^.

**Figure 6:**
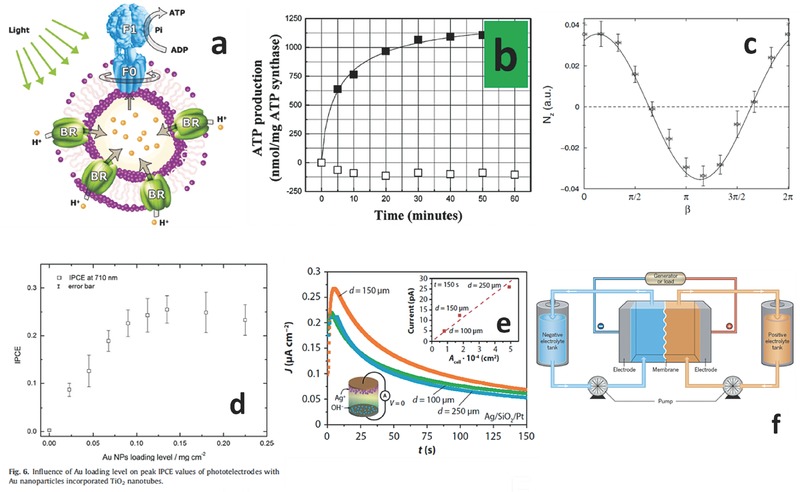
Energy generation/storage – enabling subsystems. a) Schematic representation of proteopolymersomes where ATP synthase (composed by the membrane‐integrated domain Fo and the soluble domain F1) uses an electrochemical proton gradient generated by the energy transducing protein bacteriorhodopsin (BR) to synthesize ATP from ADP and inorganic phosphate (Pi). b) Photoinduced ATP synthesis in the BR‐ATP synthase proteopolymersomes. The production per milligram of proteic complex is shown as a function of illumination time (full dots) and compared to dark reference (open dots). Reproduced with permission.^50^ Copyright 2005, ACS. c) Magnetic torque transferred to a FF in an experimental setup as a function of the phase angle β of an AC magnetic field applied along y‐axis plus a DC static magnetic field applied along x‐axis, where macroscopic torque is generated by thermal noise and Brownian motion. Reproduced with permission.^52^ Copyright 2003, APS. d) influence of Au loading level on the IPCE values of photoelectrodes with Au NPs incorporated in titania nanotubes, showing the potentiality of nanostructured systems in generating exciton couples under light stimulation. Reproduced with permission.^53^ Copyright 2015, Elsevier. e) short circuit current density measurement of a nanobattery based on a thin film stack Ag/SiO_2_/Pt, showing discharge curves at different cell sizes. Reproduced with permission.^54^ Published under CC‐BY 4.0 license 2013. Copyrighted by the authors. f) renewable liquid energy storage concept, where tanks of liquid metals or polymers represent positive and negative sides of a flow battery. Reproduced with permission.^55^ Copyright 2015, Nature Publishing Group.

A fully biomimetic approach, aimed at the bottom‐up construction of artificial organelles capable of making available bioenergy, is currently under study by researchers (**Figure** **6**a).^50^ In particular the synthesis of adenosine or guanosine triphosphate (ATP or GTP), accomplished by a number of enzymes dispersed in the glycosol and light driven, leading to quantum yields as high as 7% (Figure 6b).^51^ However, such approach poses some issues regarding the control of physical parameters of the SFS, as enzyme functionality requires pressure and temperature ranges to be carefully controlled. For example, it was reported that functionality might still be maintained after some months of storing the enzyme complex at a constant temperature of 277 K. The total amount of ATP synthesized was in the order of 4 ng/min/mg of ATP synthase. At the present time, devices able to use ATP to perform complex operations are still lacking, but one may think of a reversible functionalization linking ATP to the FF NPs, moving according to a chemical potential and performing mechanical work. ATP molecules could diffuse into the liquid matrix of the SFS in a free concentration driven process.

Very recently, researchers reported the photogeneration of a proton gradient across the membrane of a synthetic protocell of 0.061 pH units per minute, equivalent to an electromotive force of 3.6 mV min^−1^, that could be harvested to produce ATP.^56^

It has been shown experimentally, and explained theoretically, that FFs could act as thermal ratchets, when submitted to a steady and alternating magnetic field in two orthogonal directions, producing a momentum transfer and a rotation along the off‐plane axis (Figure 6c).^52^, ^57^ This system could be used to harvest energy from Brownian motion, by embedding in the fluid MEMS with integrated microcoils. It could be possible to enhance energy harvesting by transferring heat via microwaves, IR, oscillating magnetic fields, laser beams, and give enough time at the SFS to store energy in supercapacitors integrated onboard the floating ICs/MEMS.

A mechanism we propose to locally create an energy injection (a global energy transfer mechanism) is that of focusing a high‐density flash lamp on the SFS and convert the energetic component of the spectrum into exciton couples. This mechanism was widely investigated in the so‐called Dye Sensitized Solar Cells (DSSCs), by making use of different forms and crystalline structures of wide band gap semiconductors coupled to dyes, such as titanium NPs and Pt, the cheapest and most effective one.^58^ Recently, by using mesoporous oxides in the form of nanotubes coupled with noble metal NPs able to sustain Surface Plasmon Resonance (SPR), it has been possible to boost the quantum efficiency of these systems, reaching a remarkable Internal Photon Conversion Efficiency (IPCE) of 0.25 (electrons per second per photons per second at a certain wavelength) (Figure 6d).^53^ By making certain hypothesis on the aspect ratio of the oxides (tubes with a diameter of 50 nm and a length of 1 µm) and on the volume concentration in the smart fluid (5%), we observed that theoretically it is possible to successfully generate 430 µW by direct conversion of 1 W_opt_ using 1cc of liquid, given the typical operational voltage of a complete DSSC cell (around 0.3 V).

### 6.2 Energy Storage

ElectroChemical Mechanism (ECM)‐based memristors, a current trend in more‐than‐Moore electron devices research (discussed in the related section of this paper), would realize their resistive hysteresis based on a redox reaction taking place in their structure. As an example, the multilayered stack Ag/SiO_2_/Pt represents a prototypical ECM cell, where Ag in the metallic state gets oxidized generating Ag^+^ ions that react with OH^−^ available at the Pt electrode, creating a metallic Ag bridge across the oxide layer. This reaction is voltage controlled and perfectly reversible and was shown to generate electromotive forces and correspond to a charge stored in the thin film. This arrangement was called a nanobattery (Figure 6e).^54^

By computing the typical charge that can be stored in a device having a typical vertical structure composed by 30–50 nm of SiO_2_ and a typical area of 2 × 10^−4^ cm^2^, one obtains a value of ≈5 nC. Considering core‐shell NPs having an Ag 50 nm‐diameter core surrounded by a 30–50 nm of SiO_2_, in order to have the same area one would need ≈300,000 particles. Calculating an average density of the distributed nanobatteries around a volume ratio of 5%, that number of particles corresponds to a volume of about 1 × 10^−14^ cm^3^, meaning that 1 single cc of distributed nanobattery‐functionalized SFS could be potentially able to store 500,000 C. Trying to translate those numbers in more familiar Ah units, and using again available technology, in the hypothesis of being able to collect from the distributed nanobatteries all the generated current from a remote electrode, we would have the current density additively approaching to 0.1 pA × 10^14^ = 10 A, discharged in a time of ≈10,000 s (2.8 h), leading to a liquid battery capacity of ≈3.6 Ah. The drawback of this approach is the low voltage, since such ECM memristor‐like cells will have typical electromotive forces in the range of 50 mV (pretty much enough to guarantee operation of modern low‐power circuits), leading to volume energy densities of ≈180 Wh/l, higher than the standard lead‐acid cell (up to 100 Wh l^−1^) and comparable to NiCd rechargeable batteries (up to 200 Wh l^−1^). The real advantage is mass: since the very low fraction of NPs could lead to a density of 1.1 kg l^−1^, we can now compare the mass energy density of ≈160 Wh kg^−1^ with polymer Li ion cells (up to 200 Wh kg^−1^), with the additional advantage of being liquid, which is a recent trend in batterey research and the opportunity to embed other functionalities in the SFS (Figure 6f).^55^

### 6.3 Sensing Mechanisms

**Figure 7:**
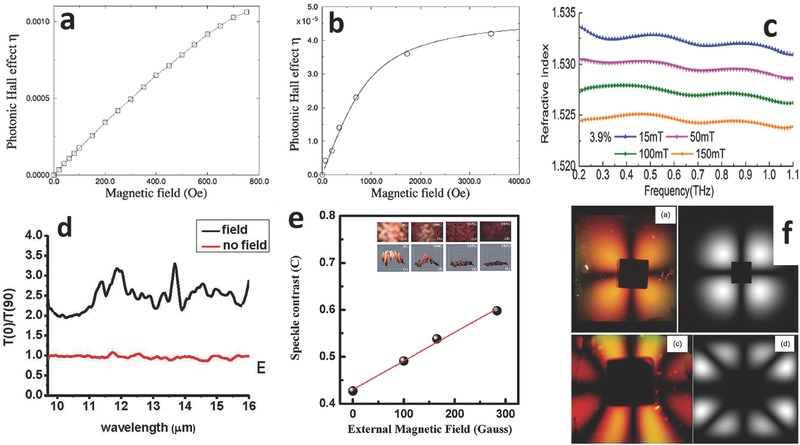
Sensing – enabling subsystems. a) Experimental measurement of the PHE generated by a sol‐gel of cobalt ferrite at low magnetic fields, with a frequency of 560 Hz. b) same as before in the high field regime and with a frequency of 40 Hz. Reproduced with permission.^60^ Copyright 2000, APS. c) theoretical refractive index spectra of a FF having 3.9% concentration under electromagnetic waves in the THz regime, showing magnetic field tenability. Reproduced with permission.^62^ Published under CC‐BY 4.0 license 2014, Optical Society of America, copyrighted by the authors. d) Extinction coefficient of FF transmissions at 0 and 90° subjected to external magnetic field, exhibiting pronounced resonances around the surfactant absorption line (≈12 µm) and the kerosene absorption line (≈14 µm), providing clear evidence of field enhancement by NP ordered chains. Reproduced with permission.^61^ Published under CC‐BY 4.0 license 2014, copyrighted by the authors. e) linear variation of speckle contrast of backscattered speckles as a function of external magnetic field, induced by Raileigh – Taylor instabilities. Reproduced with permission.^63^ Copyright 2013, AIP. f) Comparison between light patterns (left) and simulations (right) using polarized light with a FF cell, when a permanent magnet is placed in contact with the liquid in a single pole mode (top row) and dipole mode (bottom row). Reproduced with permission.^95^ Published under CC‐BY 4.0 license 2014, copyrighted by the authors.

Thanks to the strong light scattering phenomenon provided by FFs, many effects have been observed. Some of these effects are useful for magnetic field sensing, in particular the Photonic Hall Effect (PHE) and the Optical Magnetoresistance (OMR).^59^ The first one, already experimentally demonstrated, enables a highly accurate reading of the external magnetic field in a limited range.^60^ Published data report a saturation above 0.1 T, even though this limit strongly depends on the FF choice, and a quasi‐linear dependence at least down to 1 mT (**Figure** **7**a, b). Analyses in the THz regime, which is capable of exceptional composition discrimination due to inherently compound‐dependent fingerprints exhibited in this bandwidth, could represent a potentially interesting approach to enable new science in the THz regime.^61^ Experimental realization of a THz magnetically operated FF based modulator was achieved and reported (Figure 7c,d).^62^ FFs are expected to be applicable in THz sensing, modulation, phase shifting and polarization control. The Rayleigh – Taylor instability could be used to acquire information on the gravitational field: when placing a FF on top of a less viscous liquid, fingering would occur, producing a surface roughness that is a function of both the magnetic field and the gravitational one (Figure 7e, f).^63^

Ex‐situ sensors, more traditionally adapted to the Soft Robotics field, could be used too, exploiting resistive or capacitive (impeditive) changes, magnetic or optoelectronic properties. A very recent review on flexible and stretchable conventional sensors is given here.^64^

### 6.4 Information Chain Overview

The collection and distribution of information within a SFS is fundamental to achieve autonomous behavior, mimicking the biological paths from the sensory afferent nerves to the brain and from the brain to the efferent motor nerves, in biological units. The following functionalities would be required of SFSs:

- information collection from embedded sensors and distribution to active areas, in particular for mobility, through specific communication channels (wired, wireless, depending on architectural choices),
- storage of information through distributed reactions (electrochemical, phase‐change, chemical reaction) or through functional particles embedded in the SFS, such as memristors/resistive switching devices (RSDs) or standard flash memory cells embedded in floating ICs,
- information elaboration/computation, where learning could be introduced as an electrochemical or configurational state variation induced by a sequence of electrical stimuli or chemical reactions.^65^

### 6.5 Information Storage and Elaboration

**Figure 8:**
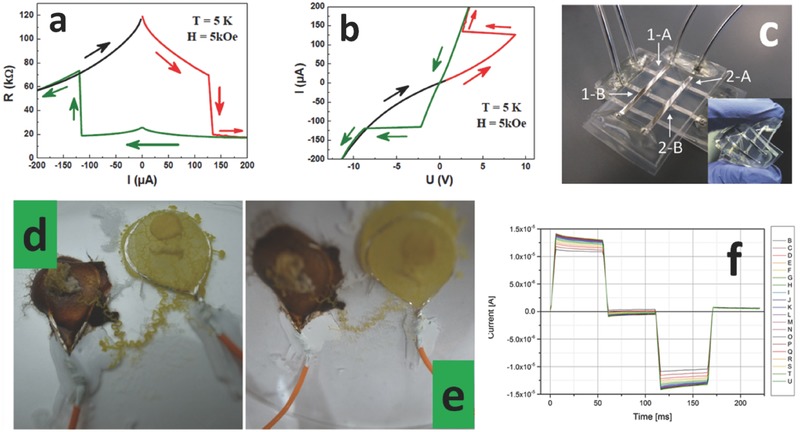
Information elaboration and storage‐enabling subsystems. a) Resistance versus current of a nanocolumnar LSMO/Al_2_O_3_/LSMO device at 5 K. b) Extrapolated IV curve in the same system as before. Reproduced with permission.^75^ Copyright 2013, the authors. c) Quasi‐liquid array of RSDs encapsulated in a PDMS microfluidic chip. Reproduced with permission.^77^ d) Physarum Polycephalum inoculated with NPs before electrical characterization. e) same as before after electrical characterization, showing connection thinning and burning out of unnecessary connections. d,e) Reproduced with permission.^76^ Copyright 2013, the authors. f) pulsed electrical characterization showing programmable‐nanobattery features in a synthetic liquid RSD. Reproduced with permission.^71^ Copyright 2016, RSC.

At the present time, Resistive Switching Devices (RSDs) are gaining significance mainly due to their prospective application in the field of digital memories.^66^ RSD can be used to perform analog computing at digital speeds. It was in 1971 when the so called memristor was mathematically proposed as passive device and subsequently realized.^67^, ^68^ RSDs are classified into organic, inorganic and hybrid, according to the nature of the active layer that is present between the two electrodes or leads.^69^, ^70^, ^71^ Recently a debate has been on‐going on the actual existence of ideal memristors or not, and also echoes from the past show that memristors had been known since 1880s.^72^, ^73^ The exponential growth of Moore's law and the fear of saturation that the microelectronic industry is about to reach, has spurred further research on this topic, opening the door of direct neuromorphic architecture implementations.^74^ Regarding the applicability of RSDs to SFS, one can see that it is extremely difficult to find published assessments of their engineering figures of merit in the low temperature limit. There is a report showing that they could work down to 5 K (**Figure** **8**a, b).^75^ Regarding the typical figures of merit that theoretically we will able to store in a single floating “smart dust” speck, in other words a typical silicon chip 100 × 100 µm^2^, and according to data diffused by HP, Co. with no commercial devices available on the shelf yet, we could have up to 1 kbit of information stored in one layer. This layer would work up to 1 GHz and down to 5 K, since it would not be based on semiconductors. It would be rad‐hard in virtue of its monocrystalline structure. Considering the computation capabilities, on the same area one could have 500 neuromorphic synapses, enough to implement basic neuromorphic sorting algorithms.

Another possible implementation of RSDs functionalities is that of distributed NP‐based computing, taking advantage of the liquid matrix. So far, no one has addressed the problem and really considered in depth how to implement a communication protocol between single agents dispersed in a liquid matrix. Experiments have been done with biological living organisms, such as *Physarum Polycephalum*, showing that they would behave as a memristor network, maintaining fluid behavior and intelligent flow capabilities (Figure 8d,e).^76^ A FF system would embody all the necessary elements to realize a similar distributed computation model: agents able to switch between different states (two or even more), controlled by means of a state variable (magnetic field) and producing effects on another variable (current) through their long range interaction (dipolar field, itinerant carriers carrying magnetic momenta). In the literature we can find rare examples of hybrid architectures, approaching a quasi‐liquid form where metal leads are realized using low melting point alloys, the housing is fabricated in PDMS, and the active part takes the form of a hydrogel (Figure 8c).^77^ Another result reports suggests that human blood behaves as a memristor, which has been confirmed by independent research from other groups.^78^ Our research group reported the first‐ever synthetic liquid memristor response based on ZnO NPs dispersed in a liquid monomer (Figure 8f).^71^

Other groups report the concept of “Digital Colloids”, in other words the capability of encoding massive amounts of information in the controlled assembly of NPs in bigger clusters, even though, as said, there is no suggestion on how to address each information node and write/extract information.^79^

### 6.6 Information Transmission

Considering the advantage offered by the liquid form of a SFS, we could think of Localized Colloidal Microantennas (LoCµAs), based on MeLLFs, localized on the external surface of the SFS. Since MeLLFs are a stabilized layer of metallic NPs positioned at the interface between two immiscible liquids, we could think of positioning small volumes of water to create liquid reflectors, with a typical diameter ranging from 100 µm to 10 cm. Their shape could be optimized by setting the total volume of SFS into vibration, using piezoresonators mounted onboard floating ICs/MEMS; this would lead to oscillation of the surface of MeLLFs in LoCµAs, reaching at some point of the oscillation the desired parabolic profile and enabling a short time transmission/reception of data.

Since the LoCµAs are adaptable antennas, by proper characterization it would be possible to perform complex analysis and/or transmissions. As low‐energy alternative, it could be possible to pin LoCµAs on plastic lenses, having fixed shape, in order not to need any oscillation to operate them. Exploring the transmission bands within FFs is fundamental to achieve data transfer between floating ICs and other active part in the SFS.

**Figure 9:**
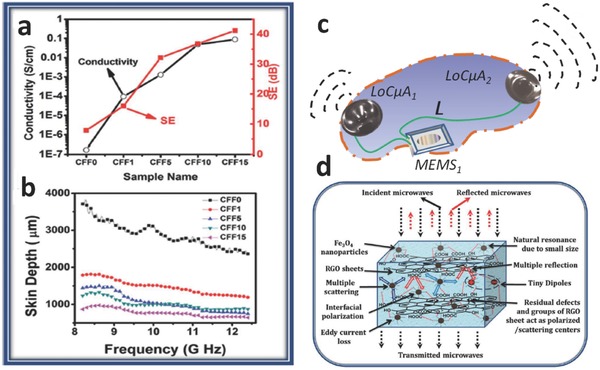
Information transmission – enabling subsystems. a) Conductivity and shielding efficiency (SE) of different liquid formulations containing reduced graphene oxide and FFs. b) skin depth measurements of the same formulations as a function of frequency in the GHz range. c) sketch showing a possible configuration for localized colloidal microantennas, where two assemblies based on MeLLFs are put into oscillation to realize transmissions and are connected through wirings to a floating MEMS/IC. d) proposed mechanisms for microwave absorption/refraction in a FF‐based formulation. Reproduced with permission.^80^ Copyright 2014, RSC.

FFs are extremely efficient microwave absorbers, their efficacy being directly related to the electrical conductivity; they can absorb up to 46 dB (99.9% attenuation) in the range 8–13 GHz (**Figure** **9**a to c).^80^, ^81^ Therefore, standard GHz communication signals should not be used to transfer information, for example from floating ICs to the exterior of the SFS.

It has been shown that liquid metal devices based on EGaIn offer also exceptional controllability in the THz regime, where it is possible to tune from wideband to dual‐band behavior and also to tune the working frequency.^82^

Regarding optical properties, it has been reported that ordered arrays of Co NPs in a FF, under the effect of a magnetic field, could create a tunable photonic hyper‐crystal, having two spatial periodicities (one corresponding to particle size, the other to the aggregated/depleted volumes).^61^ This produces a combination of two different objects: a photonic crystal with optical bandgaps and photon sub‐wavelength localization and a hyperbolic metamaterial. This property could be used to localize light, in a way similar to SPR, and perform extremely enhanced optical sensing of chemical molecules in contact with the NPs (SERS‐like sensing).

### 6.7 Mobility

**Figure 10:**
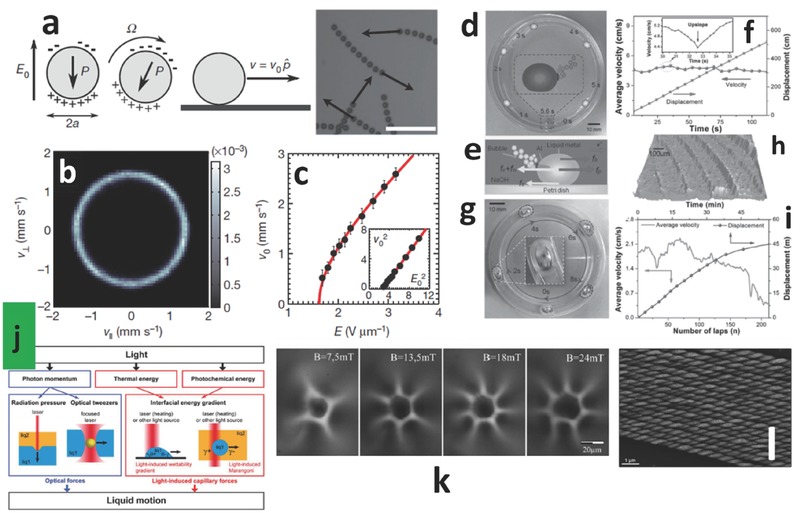
Mobility – enabling subsystems. a) Sketch of the Quincke rotation and self‐propulsion mechanism of a colloidal roller characterized by its electric polarization *P* and superposition of ten successive snapshots of colloidal rollers in a time interval of 5.6 ms, scale bar 50 µm. b) Probability distribution function of the velocity vector for isolated rollers, where the parallel direction of velocity is that corresponding to the tangent of racetrack. c) Roller velocity plotted versus electrical field amplitude. Reproduced with permission.^83^ Copyright 2013, Nature Publishing Group. d) self‐fueled liquid metal motor based on EGaIn droplet of 60 µl volume running in a circular Petri dish containing 0.25 mol l^−1^ NaOH solution. e) schematics of forces affecting the velocity of the liquid motor. f) Time‐average velocity and displacement in a 112 s observation window. g) 160 µl volume droplet running in a circular open‐top channel. h) surface topography of the circular channel. i) lap‐average velocity and time‐displacement plots in a 1 h observation window. Reproduced with permission.^89^ Copyright 2015, Wiley‐VCH. j) Main strategies to convert light stimuli into liquid motion. Reproduced with permission.^84^ Copyright 2012, RSC. k) Whirlpool structures induced in a FF using a holographic optical trap setup at different values of magnetic field. Reproduced with permission.^85^ Copyright 2015, Oficyna Wydawnicza Politechniki Wroclawskiej. l) Scanning electron microscope image showing NPs assemblies in a FF into helical superstructures. Reproduced with permission.^87^ Copyright 2015, RSC.

It has been shown that hydrodynamic interaction could promote the emergence of collective motion in a colloidal system in the form of a macroscopic flow (**Figure** **10**a to c).^83^ A relevant result in passive propulsion was obtained by means of chemical propellant, in an ionic environment, obtaining the self‐propulsion of what was called liquid metal mollusk, for more than 45 m (Figure 10d to i).^83^ Magnetodielectric small (from nano to micro) particles would be subjected to the forces of external electric, magnetic and electromagnetic fields. Using a visible/UV/IR electromagnetic wave is of particular interest, due to the enormous development of low‐energy high‐coherence and controllable light sources, even though it would be necessary to consider the unavoidable interference by the external environment. Optical guidance of the particles dispersed in a medium is possible, as vastly calculated and experimentally proven in literature, following the so‐called field of optical trapping and exploiting the collective properties of magnetic elements.^84^ The possible mechanisms to convert light into motion are: radiation pressure, optical tweezers, light induced wettability gradients, thermocapillary effect, photosensitive surfactants, chromocapillary effect (isothermal variation of surface tension, inducing a Marangoni flow), optoelectrowetting, photoelectroosmotic effect and optical dielectrophoresis (Figure 10j). Regarding the applications, in literature we may find demonstrations of the basic functions necessary to build a microfluidic device, such as injection, pumping, valves, fusion, deviation, sorting and droplet generation, but we are still far from the requirements of an autonomous externally controlled mobility system.^84^ An example is given by the formation of stable structures as collective optical vortices.^85^ It is known that a laser beam carrying an optical vortex, when submitted to a static magnetic field, is able to induce in FFs whirlpool structures, very stable and reproducible (Figure 10k). Direct optical actuation of azobenzene‐functionalized liquid crystalline polymers was achieved in structures having the shape of a cantilever, in the millimeter range.^86^ The structure was effectively bent using a 100 mW/cm^2^ radiation towards (away from) the light source using a polarization parallel (perpendicular) to the main cantilever axis. Superstructures made by a collection of simple agents, such as magnetite NPs, could be used to increase mechanical stiffness/viscosity of the colloid in an anisotropic way, by replicating biological structures such as actin filaments (Figure 10l).^87^ Peristaltic motion found in worm‐like animals could be used in those environments where a sufficiently high gravitational field would produce friction.^88^ The practical implementation of such a system could be based on mechanical buckling, induced by varying the stiffness of small hemispherical volumes positioned on the surface of the outer skin, by means of either MRF or ERF.

Motion in a non‐Newtonian liquid robot was also demonstrated using an array of acoustic transducers, producing controllable movements, and also the capability of carrying a load.^90^

Chemical reaction, involving the use of gel inks flowing in a synthetic vascular system, have been demonstrated to successfully produce mechanical expansion and soft body extension/retraction in an “octobot”, or octopus robot, exploiting the catalytic generation of oxygen on Pt‐covered surfaces from the monopropellant hydrogen peroxide, activated by controlled heating.^91^

Electrical fields have shown to successfully and reversibly elongate chains of self‐assembled Janus ellipsoids by 36%, capable of having a shape memory and representing a useful solution for the mobility of SFS.^92^

## 7 Smart Fluid System Taxonomy

**Figure 11:**
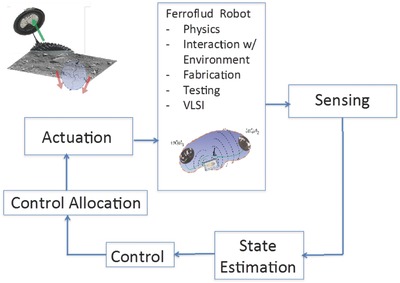
Autonomy functions of SFS. Functional block diagram of the autonomy functions of an SFS, including SFS state sensing and estimation, and feedback of the state to the control function which would enable transduction in a physical environment.

To summarize and better visualize the observations reported in this study, **Table** **1** collects the the results of a trade‐off among different types of liquid matrices for the SFS. The color code stands for a planetary exploration scenario, providing hints about suitability for space application: small robotic vehicle subject to low gravitational force and no atmosphere (green), Venus‐like robotic vehicle subject to high temperatures and pressures (red), Titan‐like robotic vehicle subject to low temperatures and high pressures (violet), gas/ice giant with huge pressures and supercritical atmosphere (blue). **Table** **2** provides a simplified scheme of the basic and advanced functionalities for a SFS. Finally, **Figure** **11** shows a functional block diagram of the proposed autonomy functions of an SFS, including SFS state sensing and estimation, and feedback of the state to the control function which would enable transduction in a physical environment.

**Table 1:**
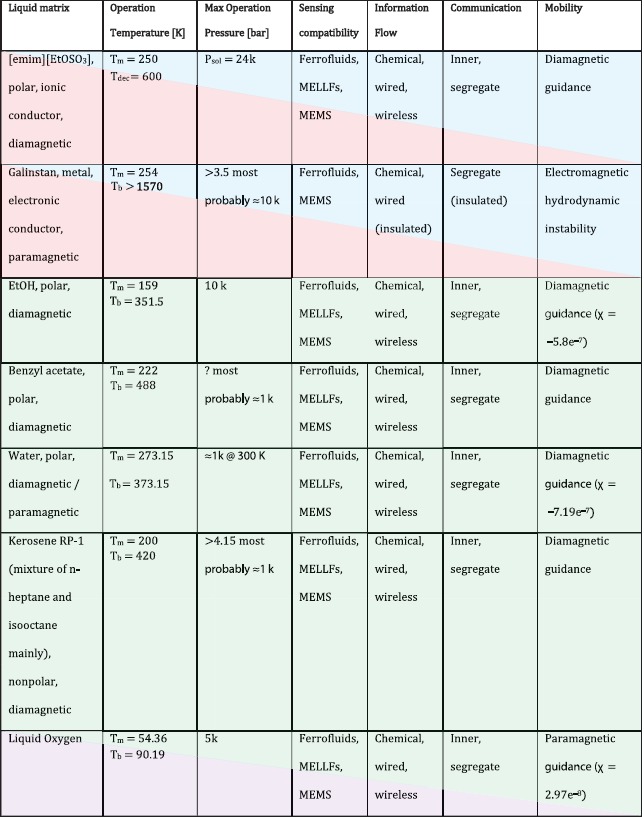
Trade‐off among different types of liquid matrices for the SFS. The color code stands for a planetary exploration scenario, providing hints about suitability for space application: small robotic vehicle subject to low gravitational force and no atmosphere (green), Venus‐like robotic vehicle subject to high temperatures and pressures (red), Titan‐like robotic vehicle subject to low temperatures and high pressures (violet), gas/ice giant with huge pressures and supercritical atmosphere (blue)

**Table 2:** Taxonomy table listing the basic and advanced functionalities for a SFS

Mechanism/Functionality	Matrix or filler to enable it	Mass/volume constraints	Energy required	Passive operation	Active operation	Pros and Cons
Omeostasis	Phase change core or active heaters	Scalable	Joule heating 10 W	Y	Y (electronic activated system)	Cons: additional mass required
Mobility	Ferrofluid	N	Unknown	Magnetotactic (poor, necessitates environmental magnetic field)	Optically pumped (slow?)	Cons: external source required
Energy harvesting	Oxide‐based exciton converters	Scalable	Artificial light source/sun (mW)	Y	N/A	Cons: complex realization
Energy storage	Onboard IC supercapacitors	Miminum die size 100 × 100 µm^2^	Charge transfer from harvester (µW)	N	Y	Pros: high energy density
Pressure sensing	Ferrofluid and magnetic susceptibility measurement	Miminum die size 100 × 100 µm^2^	Impedance measurement (µW)	N	Y	Pros: integrated with temperature data
Temperature sensing	Ferrofluid and magnetic susceptibility measurement	Miminum die size 100 × 100 µm^2^	Impedance measurement (µW)	N	Y	Pros: integrated with pressure data
Magnetic field sensing	Ferrofluid and optical reading (PHE)	Miminum die size 100 × 100 µm^2^	Optical reading through laser (mW)	N	Y	Cons: it works down to the mT range
Chemical sensing	Ferrofluid and THz reading	Miminum die size 100 × 100 µm^2^	Reading through THz device (mW)	N	Y	Cons: requires an opening mechanism to let molecules diffuse and bind to the ferrofluid
Optical imaging	Solid state devices on the outer skin	Minimum assembly size 1 × 1 mm^2^	100 mW	N	Y	
Data storage	Memristors integrated on ICs	Miminum die size 100 × 100 µm^2^	Storage: mW Reading: nW	N	Y	Pros: stable with temperature and rad‐hard
Data elaboration	Memristors integrated on ICs	Miminum die size 100 × 100 µm^2^	Storage: mW Reading: nW	N	Y	Pros: stable with temperature and rad‐hard
Data transmission	MELLFs in LoCµAs	Miminum LoCµA size 1 × 1 mm^2^	Transmission: mW depending on the range	N	Y	

## 8 Future Applications

Different scenarios of application would be possible for SFS, and they are discussed next.

Smart fluid as in‐situ actuator (mobility for locomotion): in one scenario, the smart fluid could be an element of a mobility system. Instead of operating as a wheel, the smart fluid would be contained inside a membrane, and would provide lift and propulsion (upwards and forward thrust) capabilities against the environment to a surface where the scientific payload of interest is located.

Smart fluid as in‐situ sensor (in‐situ science data gathering): in another scenario, the SFS would operate, collectively, as an optofluidic magnetometer, thanks to the magnetic dipole distribution in the colloidal suspension of MEMS.

Smart fluid as energy barrier/harvester: in another scenario, the smart liquid device would reconfigure itself to provide protection to extreme temperatures, radiation, wind, illumination, not only for other robotic assets, but possibly also for human agents. Similarly, in another scenario, the SFS would have an energy harvesting capability via a selective patch, capable of relocating itself to a more efficient energy‐harvesting location where a photo‐induced chemical process would take place, to charge an energy storage unit.

More practically future application of SFS could be terrestrial ones. They could be components of industrial assembly lines or waste removal lines; exploited in search and rescue applications, percolating and dripping in areas where access is limited, such as post‐earthquake or post‐avalance scenarios. The scenario of highest commercial relevance would be that of wearable technologies,^93^ where the advent of SFS could bring tremendous benefits, such as energy harvesting from human body heat, sensing, protection from radiation/electromagnetic background/extreme temperatures, motion aid and control co‐robotics, where the garment could behave as a *second skin*.^3^ In parallel, we envisage that SFS could be exploited in medical applications for drug delivery, as distributed internal “repair kit” and to provide a mechanism for in situ diagnosis.

## 9 Conclusions

Smart Fluid Systems are defined as devices based on organic or inorganic liquid, contained inside a volume by surface tension or by a confining membrane that protects them from a harsh planetary environment. In a biologically inspired vision, SFS could be able of changing shape according to a specific command or by means of a fully passive adaptive system, and provide a solution for innovative space exploration applications in extreme or otherwise challenging environments. Such a liquid robotic system would have the potential of offering innovative solutions to mobility, sensing, energy‐harvesting, and as energy barrier. Until now, research in this area has provided well established proofs of the feasibility of development of a SFS, although no experiment has been done yet in the direction of having a complete autonomous system. The scope of this review was to provide a strong foundational support to build the new field of liquid robotics.

## Conflict of Interest

The authors declare no conflict of interest.
